# Whole-genome analysis of rotavirus G4P[6] strains isolated from Korean neonates: association of Korean neonates and rotavirus P[6] genotypes

**DOI:** 10.1186/s13099-019-0318-5

**Published:** 2019-07-10

**Authors:** Su-Kyung Lee, Seoheui Choi, Jae-Seok Kim, Eun Jin Lee, Jungwon Hyun, Hyun Soo Kim

**Affiliations:** 1 Department of Laboratory Medicine, Hallym University Dongtan Sacred Heart Hospital, College of Medicine, Hallym University, 7, Keunjaebong-gil, Hwaseong-si, Gyeonggi-Do, 18450 South Korea; 2 Department of Pediatrics, Hallym University Dongtan Sacred Heart Hospital, College of Medicine, Hallym University, 7, Keunjaebong-gil, Hwaseong-si, Gyeonggi-Do, 18450 South Korea; 3 Department of Laboratory Medicine, Kangdong Sacred Heart Hospital, College of Medicine, Hallym University, 150, Seongan-ro, Gangdong-gu, Seoul, 05355 South Korea

**Keywords:** Rotavirus, Genotype, G4P[6], Korean, Neonate

## Abstract

**Background:**

Group A rotaviruses are the major causative agents of pediatric gastroenteritis worldwide. Several studies have reported the predominance of G4P[6] rotavirus genotypes in Korean neonates, which is uncommon in other countries. Therefore, the purposes of this study were to determine the genotype constellations of complete genomes of G4P[6] rotavirus strains isolated from Korean neonates using next-generation sequencing, to compare these sequences with other G4P[6] strains in other countries, and to determine the reason for the predominance of G4P[6] genotypes in Korean neonates.

**Results:**

Twenty rotavirus G4P[6] strains, isolated from January 2013 to January 2016, were selected for whole-genome sequencing. Eleven rotavirus genes were amplified using specific primer sets, and sequencing was carried out using an Ion S5 XL next-generation sequencing platform. Genotypes of each gene were determined, and phylogenetic analyses were performed to investigate genetic distances between genes of rotaviruses in this study and those of other rotavirus G4P[6] strains whose whole-genome sequences were previously published. All 20 rotavirus strains in this study had the same genotype: G4-P[6]-I1-R1-C1-M1-A1-N1-T1-E1-H1, representing the Wa-like genotype constellation. BLAST searches of 20 G4P[6] rotavirus strains revealed that all G4 sequences in this study showed the highest nucleotide identity to G4 sequences of G4P[6] rotavirus strains isolated in Korea in 2008 (GenBank accession number: FJ603447). Additionally, P[6] gene sequences in this study showed the highest nucleotide identity to P[6] sequences of G4P[6] strains detected in Korea in 2002 (AY158093). Phylogenetic and nucleotide sequence analyses showed that G4P[6] strains in this study and previously reported G4P[6] strains in Korea were mostly detected in neonates and had similar G4 and P[6] sequences compared with other G4P[6] strains detected in other countries.

**Conclusions:**

This study showed that the whole-genome constellation of rotavirus G4P[6] strains from Korean neonates resembled a Wa-like genotype constellation. Additionally, rotavirus genotypes detected in Korean neonates had unique P[6] sequences, which may be the cause of Korean neonatal rotavirus infection.

**Electronic supplementary material:**

The online version of this article (10.1186/s13099-019-0318-5) contains supplementary material, which is available to authorized users.

## Background

Group A rotaviruses are the most important pathogens causing pediatric gastroenteritis worldwide. The virus contains a triple-layered capsid surrounding a genome of 11 double-stranded RNA segments [1]. The outer capsid layer is composed of two structural proteins, VP7 and VP4, which are targets of neutralizing antibodies. Based on VP7 and VP4 gene sequences, human group A rotaviruses are classified into G and P genotypes, and an epidemiological study showed that at least 35 G and 50 P genotypes exist [1, 2]. G1–G4 and G9 and P[4], P[6], and P[8] are the most frequent G and P genotypes, respectively [1, 3], and the genotypic distribution of rotavirus strains shows temporal and geographical fluctuations [1, 4]. In addition to VP4 and VP7 genotyping, a complete genome classification system was developed by the Rotavirus Classification Working Group [5]. The genotypes of the VP7–VP4–VP6–VP1–VP2–VP3–NSP1–NSP2–NSP3–NSP4–NSP5/6 genes of each rotavirus strain can be expressed as Gx-Px-Ix-Rx-Cx-Mx-Ax-Nx-Tx-Ex-Hx (where x represents the genotype number) to represent a genotype constellation. Most rotavirus strains detected in humans can be classified into two major and one minor genotype constellations, the Wa-like, DS-1-like, and AU-1-like genotype constellations, which are described as G1/3/4/9/12-P[8]-I1-R1-C1-M1-A1-N1-T1-E1-H1, G2-P[4]-I2-R2-C2-M2-A2-N2-T2-E2-H2, and G3-P[9]-I3-R3-C3-M3-A3-N3-T3-E3-H3, respectively; however, intergenotype reassortment events occasionally occur between strains [6–8].

In Korea, G1P[8] is the most frequent genotype in children, and G4P[6] is the most frequent genotype in neonates [3, 9–12]. The predominance of the G4P[6] genotype in Korean neonates has been frequently reported in several studies from 1999 to 2016 [9–13]. Interestingly, the predominance of G4P[6] in neonates has not been reported in other countries.

Next-generation sequencing (NGS) technology has recently been applied to viral genome research and human genome research [14]. NGS can generate large amounts of viral sequence data simultaneously within a short time through massively parallel sequencing. NGS technology reduces time, effort, and cost compared with conventional Sanger sequencing techniques, particularly when sequencing many genes or dealing with large numbers of samples.

In this study, we attempted to determine the genotype constellation of the complete genome of G4P[6] rotavirus strains characteristically isolated from Korean neonates using NGS and to compare the sequences of Korean G4P[6] strains with G4P[6] strains in other countries. In addition, we tried to determine the reason for the predominance of G4P[6] genotypes in Korean neonates.

## Results

### Genotype constellations of rotaviruses in neonates

Genotype constellations of the rotaviruses isolated from 20 neonates in this study were all the same (G4-P[6]-I1-R1-C1-M1-A1-N1-T1-E1-H1, a Wa-like genotype constellation). BLAST searches of 20 G4P[6] rotavirus strains revealed that all G4 sequences in this study showed the highest nucleotide identity to G4 sequences of G4P[6] rotavirus strains isolated in Korea in 2008 (GenBank accession number: FJ603447; Table 1). P[6] gene sequences in this study showed the highest nucleotide identity to P[6] sequences of G4P[6] strains detected in Korea in 2002 (AY158093). The GenBank accession numbers of the strains with highest nucleotide identity to the I1, R1, C1, M1, A1, N1, T1, E1, and H1 genes of most G4P[6] strains other than RN-019 identified in this study using BLAST were KJ752030, JQ863309, KT223476, KT694941, KC579614, JQ863316, LC205211, AF260930, KC580600, and AB091353, respectively (Table 1). The closest sequences to strain RN-019 detected in 2016 were LC105194 in VP6, KP645333 in VP1, LC105207 in VP2, LC105208 in VP3, LC105209 in NSP1, LC105210 in NSP2, LC105211 in NSP3, KP645342 in NSP4, and KP645343 in NSP5/6 (Table 1).

**Table 1:** Closest nucleotide sequences of 11 rotavirus genes identified using NCBI nucleotide BLAST

Gene	Specimen no.	Closest sequence at NCBI	% identity with the closest sequence	Description of the closest sequence
VP7	RN-001–020	FJ603447	99	GILL3-8/KOR/2008/G4P[6]
VP4	RN-001–020	AY158093	96	SG2 KRV01/KOR/2002/G2P[6]
VP6	RN-001–018, RN-020	KJ752030	97	Human-wt//ETH/MRC-DPRU1843/2009/G1P[8]
	RN-019	LC105194	100	Human-wt/JPN/MU14-18/2014/G1P[8]
VP1	RN-001–018, RN-020	JQ863309	96–97	Human-tc/IDN/57M/1980/G4P[10]
	RN-019	KP645333	99	Human-wt/AUS/2011/G1P[8]
VP2	RN-001–018, RN-020	KT223476	98	Human-wt/BEL/2002/G1P[8]
	RN-019	LC105207	99	Human-wt/JPN/MU14-18/2014/G1P[8]
VP3	RN-001–018, RN-020	KT694941	96	Human-wt/USA/Wa/1974/G1P[8]
	RN-019	LC105208	99	Human-wt/JPN/MU14-19/2014/G1P[8]
NSP1	RN-001–018, RN-020	KC579614	95	Human-wt/USA/DC1164/1978/G1P[8]
	RN-019	LC105209	99	Human-wt/JPN/MU14-19/2014/G1P[8]
NSP2	RN-001–018, RN-020	JQ863316	99	Human-tc/IDN/57M/1980/G4P[10]
	RN-019	LC105210	99	Human-wt/JPN/MU14-19/2014/G1P[8]
NSP3	RN-001–020	LC105211	99–100	Human-wt/JPN/MU14-19/2014/G1P[8]
NSP4	RN-001–018, RN-020	AF260930	97	94’SZ1/China
	RN-019	KP645342	99	Human-wt/AUS/CK00110/2011/G1P[8]
NSP5/6	RN-001, 002, 007, 008, 009, 010, 018	KC580600	97	Human-wt/USA/DC2956/1988/G1P[8]
	RN-003, 004, 005, 006, 011, 012, 013, 014, 015, 016, 017, 020	AB091353	97	Human/87H134/G1
	RN-019	KP645343	100	Human-wt/AUS/CK00110/2011/G1P[8]

*NCBI* National Center for Biotechnology Information, *BLAST* Basic Local Alignment Search Tool

### Phylogenetic and nucleotide sequence analyses of rotavirus G4P[6] strains

**Fig. 1:**
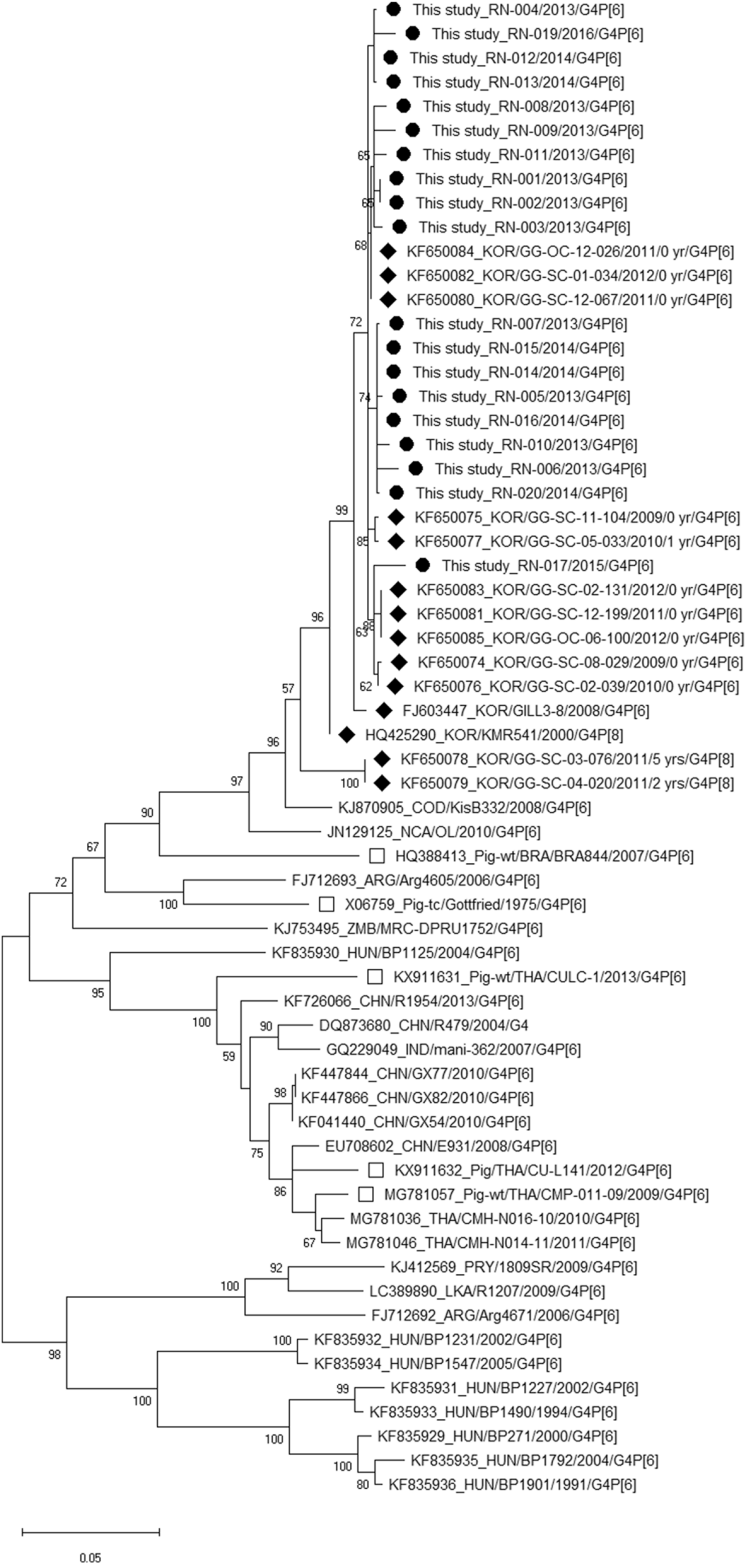
Phylogenetic tree of VP7 (G4) sequences of rotavirus G4P[6] strains in this study and other G4P[6] rotavirus strains with full genome sequences. Black circles indicate the G4P[6] strains isolated from neonates in this study, black diamonds indicate the Korean G4P[6] strains from GenBank, and empty squares indicate the porcine G4P[6] strains from GenBank

**Fig. 2:**
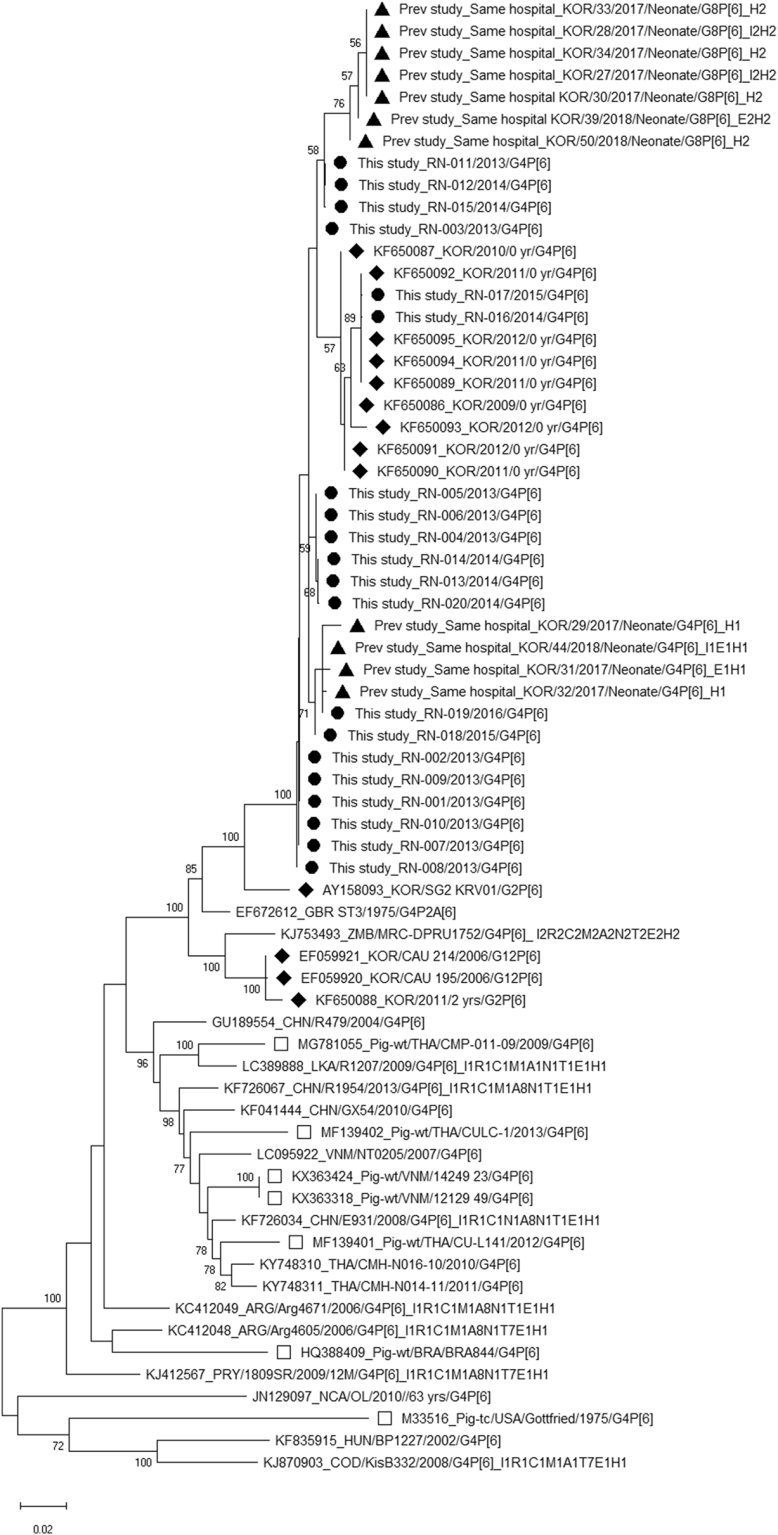
Phylogenetic analysis of VP4 (P[6]) sequences of rotavirus G4P[6] strains in this study and other G4P[6] rotavirus strains with full genome sequences. Black circles indicate the G4P[6] strains isolated from neonates in this study, black triangles indicate the G4P[6] or G8P[6] strains isolated from neonates in the same hospital in a previous study, black diamonds indicate the Korean P[6] strains from GenBank, and empty squares indicate the porcine G4P[6] strains from GenBank

Table 2, Figs. 1 (VP7) and 2 (VP4), and Additional file 1: Fig. S1, Additional file 2: Fig. S2, Additional file 3: Fig. S3, Additional file 4: Fig. S4, Additional file 5: Fig. S5, Additional file 6: Fig. S6, Additional file 7: Fig. S7, Additional file 8: Fig. S8 and Additional file 9: Fig. S9 (VP6, VP1, VP2, VP3, NSP1, NSP2, NSP3, NSP4, and NSP5) show the genetic distances of the strains in this study and other reported G4P[6] strains whose whole-genome sequences were previously published. All 20 G4P[6] strains in this study were entirely composed of Wa-like genotypes, but some G4P[6] strains with Wa-like constellations in other countries have been found to show changes in the VP6 (I5), NSP1 (A8), and NSP3 (T7) genes (values in italics in Table 2). Our G4P[6] strains and previously reported G4P[6] strains in Korea were mostly detected in neonates and had similar G4 and P[6] sequences compared with other G4P[6] strains detected in other countries or porcine G4P[6] strains (Figs. 1, 2). Recently reported G8P[6] strains detected in neonates in the same Korean hospital sampled in a previous study [15] also had similar P[6] sequences (Fig. 2). Strain RN-019 detected in 2016 showed slightly different positions in phylogenetic trees from the other strains in this study for the I1, R1, C1, M1, A1, N1, T1, E1, and H1 genes (Table 2, Additional file 1: Fig. S1, Additional file 2: Fig. S2, Additional file 3: Fig. S3, Additional file 4: Fig. S4, Additional file 5: Fig. S5, Additional file 6: Fig. S6, Additional file 7: Fig. S7, Additional file 8: Fig. S8 and Additional file 9: Fig. S9). The G4 and P[6] sequences of RN-019 showed high identity (99%) with those of the other strains (RN-001–018, RN-020); however, the I1, R1, C1, M1, A1, N1, T1, E1, and H1 genes of RN-019 were found to be less similar to those of the other strains: I1 (95%), R1 (93%), C1 (93%), M1 (91%), A1 (84%), N1 (92%), T1 (95%), E1 (93%), and H1 (96%) (Table 2, Additional file 1: Fig. S1, Additional file 2: Fig. S2, Additional file 3: Fig. S3, Additional file 4: Fig. S4, Additional file 5: Fig. S5, Additional file 6: Fig. S6, Additional file 7: Fig. S7, Additional file 8: Fig. S8 and Additional file 9: Fig. S9).

**Table 2:** Rotavirus genotype constellations and nucleotide sequence identities of each gene of G4P[6] rotaviruses detected in this study and G4P[6] strains reported in other countries and Korean P[6] strains with whole-genome sequences

Strains	Genotype (% identity with the same gene of this study RN-001)										
	VP7	VP4	VP6	VP1	VP2	VP3	NSP1	NSP2	NSP3	NSP4	NSP5/6
This study RN-001 2013 G4P[6]	G4 (100)	P[6] (100)	I1 (100)	R1 (100)	C1 (100)	M1 (100)	A1 (100)	N1 (100)	T1 (100)	E1 (100)	H1 (100)
This study RN-002 2013 G4P[6]	G4 (100)	P[6] (100)	I1 (99)	R1 (99)	C1 (100)	M1 (100)	A1 (100)	N1 (99)	T1 (100)	E1 (100)	H1 (100)
This study RN-003 2013 G4P[6]	G4 (99)	P[6] (99)	I1 (100)	R1 (99)	C1 (99)	M1 (99)	A1 (99)	N1 (99)	T1 (99)	E1 (99)	H1 (99)
This study RN-004 2013 G4P[6]	G4 (99)	P[6] (99)	I1 (100)	R1 (99)	C1 (99)	M1 (99)	A1 (99)	N1 (99)	T1 (99)	E1 (100)	H1 (99)
This study RN-005 2013 G4P[6]	G4 (99)	P[6] (99)	I1 (100)	R1 (99)	C1 (99)	M1 (99)	A1 (99)	N1 (99)	T1 (99)	E1 (100)	H1 (99)
This study RN-006 2013 G4P[6]	G4 (98)	P[6] (99)	I1 (100)	R1 (99)	C1 (99)	M1 (99)	A1 (99)	N1 (99)	T1 (99)	E1 (100)	H1 (99)
This study RN-007 2013 G4P[6]	G4 (99)	P[6] (99)	I1 (100)	R1 (99)	C1 (100)	M1 (99)	A1 (99)	N1 (100)	T1 (100)	E1 (100)	H1 (99)
This study RN-008 2013 G4P[6]	G4 (99)	P[6] (99)	I1 (100)	R1 (99)	C1 (100)	M1 (99)	A1 (100)	N1 (99)	T1 (99)	E1 (100)	H1 (99)
This study RN-009 2013 G4P[6]	G4 (99)	P[6] (100)	I1 (100)	R1 (99)	C1 (100)	M1 (100)	A1 (100)	N1 (100)	T1 (100)	E1 (100)	H1 (100)
This study RN-010 2013 G4P[6]	G4 (99)	P[6] (99)	I1 (100)	R1 (99)	C1 (100)	M1 (99)	A1 (100)	N1 (99)	T1 (99)	E1 (100)	H1 (99)
This study RN-011 2013 G4P[6]	G4 (99)	P[6] (99)	I1 (100)	R1 (99)	C1 (99)	M1 (99)	A1 (99)	N1 (99)	T1 (99)	E1 (99)	H1 (99)
This study RN-012 2014 G4P[6]	G4 (99)	P[6] (99)	I1 (100)	R1 (99)	C1 (99)	M1 (99)	A1 (99)	N1 (99)	T1 (99)	E1 (99)	H1 (99)
This study RN-013 2014 G4P[6]	G4 (99)	P[6] (99)	I1 (99)	R1 (99)	C1 (99)	M1 (99)	A1 (99)	N1 (99)	T1 (99)	E1 (100)	H1 (99)
This study RN-014 2014 G4P[6]	G4 (99)	P[6] (99)	I1 (99)	R1 (99)	C1 (99)	M1 (99)	A1 (99)	N1 (99)	T1 (99)	E1 (100)	H1 (99)
This study RN-015 2014 G4P[6]	G4 (99)	P[6] (99)	I1 (99)	R1 (99)	C1 (99)	M1 (99)	A1 (99)	N1 (99)	T1 (99)	E1 (99)	H1 (99)
This study RN-016 2013 G4P[6]	G4 (99)	P[6] (98)	I1 (99)	R1 (99)	C1 (99)	M1 (99)	A1 (99)	N1 (99)	T1 (99)	E1 (99)	H1 (99)
This study RN-017 2015 G4P[6]	G4 (99)	P[6] (98)	I1 (99)	R1 (99)	C1 (99)	M1 (99)	A1 (99)	N1 (99)	T1 (99)	E1 (99)	H1 (99)
This study RN-018 2015 G4P[6]	G4 (98)	P[6] (99)	I1 (99)	R1 (99)	C1 (99)	M1 (99)	A1 (99)	N1 (99)	T1 (99)	E1 (99)	H1 (100)
This study RN-019 2016 G4P[6]	G4 (99)	P[6] (99)	I1 (95)	R1 (93)	C1 (93)	M1 (91)	A1 (84)	N1 (92)	T1 (95)	E1 (93)	H1 (96)
This study RN-020 2014 G4P[6]	G4 (99)	P[6] (99)	I1 (99)	R1 (99)	C1 (99)	M1 (99)	A1 (99)	N1 (99)	T1 (99)	E1 (100)	H1 (99)
RVA/Human tc/GBR/ST3/1975/G4P2A[6]	G4 (96)	P[6] (95)	I1 (96)	R1 (94)	C1 (94)	M1 (91)	A1 (84)	N1 (90)	T1 (95)	E1 (95)	H1 (96)
RVA/Human wt/CHN/E931/2008/G4P[6]	G4 (86)	P[6] (90)	I1 (91)	R1 (88)	C1 (88)	M1 (85)	A8 (78)	N1 (89)	T1 (89)	E1 (94)	H1 (94)
RVA/Human wt/CHN/GX54/2010/G4P[6]	G4 (87)	P[6] (91)	I1 (91)	R1 (87)	C1 (87)	M1 (87)	A8 (77)	N1 (88)	T1 (90)	E1 (93)	H1 (93)
RVA/Human wt/CHN/GX77/2010/G4P[6]	G4 (87)	P[6] (91)	I1 (91)	R1 (87)	C1 (87)	M1 (87)	A8 (77)	N1 (88)	T1 (90)	E1 (93)	H1 (93)
RVA/Human wt/CHN/GX82/2010/G4P[6]	G4 (87)	P[6] (91)	I1 (91)	R1 (87)	C1 (87)	M1 (87)	A8 (77)	N1 (88)	T1 (90)	E1 (93)	H1 (93)
RVA/Human wt/CHN/R479/2004/G4P[6]	G4 (86)	P[6] (91)	I5 (84)	R1 (88)	C1 (89)	M1 (87)	A1 (80)	N1 (89)	T7 (84)	E1 (91)	H1 (91)
RVA/Human wt/CHN/R1954/2013/G4P[6]	G4 (87)	P[6] (91)	I1 (91)	R1 (88)	C1 (88)	M1 (87)	A8 (77)	N1 (91)	T1 (88)	E1 (93)	H1 (93)
RVA/Human wt/THA/CMH-N014-11/2011/G4P[6]	G4 (86)	P[6] (90)	I5 (84)	R1 (85)	C1 (88)	M1 (85)	A8 (76)	N1 (89)	T1 (88)	E1 (90)	H1 (96)
RVA/Human wt/THA/CMH-N016-10/2010/G4P[6]	G4 (86)	P[6] (90)	I1 (88)	R1 (86)	C1 (88)	M1 (86)	A8 (76)	N1 (89)	T1 (87)	E1 (91)	H1 (96)
RVA/Human-wt/LKA/R1207/2009/G4P[6]	G4 (84)	P[6] (91)	I1 (91)	R1 (86)	C1 (88)	M1 (87)	A1 (81)	N1 (93)	T1 (89)	E1 (89)	H1 (94)
RVA/Human wt/IND/mani 362/2007/G4P[6]	G4 (85)	P[6] (87)	I1 (90)	R1 (85)	C1 (85)	M1	A8 (77)	N1 (91)	T1 (93)	E1 (94)	H1 (96)
RVA/Human wt/ARG/Arg4605/2006/G4P[6]	G4 (87)	P[6] (90)	I1 (90)	R1 (85)	C1 (88)	M1 (88)	A8 (78)	N1 (89)	T7 (82)	E1 (91)	H1 (91)
RVA/Human wt/ARG/Arg4671/2006/G4P[6]	G4 (83)	P[6] (91)	I1 (90)	R1 (87)	C1 (87)	M1 (88)	A8 (79)	N1 (89)	T1 (87)	E1 (91)	H1 (91)
RVA/Human wt/HUN/BP271/2000/G4P[6]	G4 (83)	P[6] (86)	I1 (83)	R1 (87)	C1 (87)	M1 (88)	A8 (77)	N1 (87)	T7 (83)	E1 (90)	H1 (90)
RVA/Human wt/HUN/BP1125/2004/G4P[6]	G4 (85)	P[6] (85)	I1 (85)	R1 (87)	C1 (88)	M1 (88)	A8 (77)	N1 (90)	T7 (89)	E1 (89)	H1 (89)
RVA/Human wt/HUN/BP1227/2002/G4P[6]	G4 (84)	P[6] (85)	I1 (92)	R1 (87)	C1 (88)	M1 (88)	A1 (81)	N1 (90)	T1 (89)	E1 (89)	H1 (95)
RVA/Human wt/HUN/BP1231/2002/G4P[6]	G4 (84)	P[6] (86)	I1 (91)	R1 (87)	C1 (88)	M1 (88)	A1 (81)	N1 (92)	T1 (88)	E1 (89)	H1 (95)
RVA/Human wt/HUN/BP1490/1994/G4P[6]	G4 (84)	P[6] (85)	I1 (91)	R1 (88)	C1 (88)	M1 (89)	A1 (80)	N1 (89)	T7 (83)	E1 (90)	H1 (90)
RVA/Human wt/HUN/BP1547/2005/G4P[6]	G4 (84)	P[6] (85)	I5 (84)	R1 (87)	C1 (87)	M1 (87)	A8 (76)	N1 (90)	T7 (82)	E1 (89)	H1 (89)
RVA/Human wt/HUN/BP1792/2004/G4P[6]	G4 (82)	P[6] (86)	I1 (91)	R1 (87)	C1 (87)	M1 (89)	A1 (81)	N1 (91)	T7 (83)	E1 (89)	H1 (89)
RVA/Human wt/HUN/BP1901/1991/G4P[6]	G4 (82)	P[6] (85)	I1 (91)	R1 (87)	C1 (88)	M1 (89)	A1 (81)	N1 (89)	T7 (82)	E1 (90)	H1 (90)
RVA/Human wt/COD/KisB332/2008/G4P[6]	G4 (96)	P[6] (86)	I1 (89)	R1 (85)	C1 (88)	M1 (88)	A1 (85)	N1 (90)	T7 (83)	E1 (89)	H1 (89)
RVA/Human/NCA/OL/2010/G4P[6]	G4 (94)	P[6] (84)	I1 (89)	R1 (85)	C1 (89)	M1 (85)	A8 (77)	N1 (88)	T7 (83)	E1 (87)	H1 (87)
RVA/Human wt/PRY/1809SR/2009/G4P[6]	G4 (83)	P[6] (90)	I1 (91)	R1 (87)	C1 (87)	M1 (89)	A8 (79)	N1 (88)	T7 (83)	E1 (92)	H1 (92)
RVA/Human-wt/ZMB/MRC-DPRU1752/G4P[6]	G4 (87)	P[6] (93)	I2 (78)	R2 (79)	C2 (81)	M2 (85)	A2 (76)	N2 (82)	T2 (78)	E2 (82)	H2 (84)
RVA/Human wt/KOR/CAU 195/2006/G12P[6]	G12 (73)	P[6] (93)	I1 (96)	R1 (93)	C1 (92)	M1 (91)	A1 (84)	N1 (92)	T1 (94)	E1 (93)	H1 (96)
RVA/Human wt/KOR/CAU 214/2006/G12P[6]	G12 (72)	P[6] (93)	I1 (96)	R1 (93)	C1 (92)	M1 (91)	A1 (84)	N1 (92)	T1 (94)	E1 (93)	H1 (96)

## Discussion

In this study, we performed whole genome sequencing of 20 rotavirus G4P[6] strains isolated from Korean neonates, and all 20 G4P[6] strains showed G4-P[6]-I1-R1-C1-M1-A1-N1-T1-E1-H1, a Wa-like genotype constellation. The G4P[6] strain has been frequently isolated in Korean neonates since 1999, but is rare in other countries [9–13]. Rotavirus G4P[6]/G4P[x]/GxP[6] genotypes were identified in 100% of neonatal cases at a hospital in Guri, Korea (2001–2003), 100% at a hospital in Busan (2013), 92.6% at a hospital in Seoul (2011–2012), and 93.7% of neonatal cases at a hospital in Hwaseong, Korea (2013) [9–13]. Because only the VP7 and VP4 genes of G4P[6] strains have been analyzed previously, this is the first study to analyze whole-genome sequences of all 11 genes of G4P[6] strains detected in Korea, particularly in Korean neonates. The genotype constellation, G4-P[6]-I1-R1-C1-M1-A1-N1-T1-E1-H1, found in this study was also detected in Sri Lanka in 2009, in Hungary in 2002, and in Italy in 2017 (Table 2) [16, 17]. However, neither case involved neonates. One case in Sri Lanka was a 12-month-old boy, the case in Hungary provided no age information, and the last case in Italy was a 6-month-old boy.

We performed nucleotide sequence and phylogenetic analyses of the genotypes G4, P[6], I1, R1, C1, M1, A1, N1, T1, E1, and H1 among the strains in this study and previously reported G4P[6] strains with whole-genome sequences registered in GenBank [8, 10, 18]. For the 20 strains reported in this study, there were 98–100% sequence similarities among the same genes in all 20 rotavirus strains. However, there were 82–96% sequence similarities between the G4 gene of the RN-001 sample and the G4 genes detected in other countries. There were also lower sequence similarities of 84–95% for P[6], 83–96% for I1, 85–94% for R1, 85–94% for C1, 85–91% for M1, 80–84% for A1, 88–93% for N1, 88–95% for T1, 87–95% for E1, and 87–96% for H1 compared with those in this study (Table 2). In contrast, the G4 and P[6] genotypes in this study were more similar to the G4 and G[6] genotypes previously reported in Korea. Interestingly, we recently reported that G8P[6] genotypes were also found in neonates in the neonatal intensive care unit of the same hospital as this study [15]. These P[6] nucleotide sequences of G8P[6] genotypes were not different from the P[6] gene sequences of the G4P[6] strains in this study, and these P[6] sequences of G8P[6] and G4P[6] strains also showed higher identity with the nucleotide P[6] gene sequences of G12P[6] and G2P[6] strains (GenBank no. AY158093) in Korea [18] than with the P[6] sequences in G4P[6] strains detected in other countries (Fig. 2). We investigated whether the G4P[6] strains in this study were related to porcine G4P[6] strains because several papers have provided molecular evidence that many G4P[6] strains are human–porcine RVA reassortants or even porcine RVA having directly infected children [19–21]. All G4 sequences of G4P[6] strains in this study were more similar to G4 sequences of Korean G4P[6] or G4P[8] strains in previous studies than to G4 sequences of G4P[6] strains in other countries or porcine G4P[6] strains (Fig. 1). Similarly, all P[6] sequences of G4P[6] strains in this study were more similar to P[6] sequences of Korean G4P[6], G8P[6], or G12P[6] strains in previous studies than to P[6] sequences of G4P[6] strains in other countries or porcine G4P[6] strains (Fig. 2). Therefore, the G4P[6] strains reported since 1999 in Korea can be considered endemic G4P[6] strains in Korea, not strains imported from other countries. In addition, analyses of the VP6, NSP4, and NSP5/6 genes of G8P[6] strains in a previous study showed I2, E2, and H2 genotypes, indicating the DS-1-like constellation rather than the Wa-like constellation [15]. Therefore, these new rotavirus G8P[6] strains in Korea were estimated to be derived from reassortment events between the G8-P[8]-I2-R2-C2-M2-A2-N2-T2-E2-H2 strains imported from the Asian region and the P[6] gene of endemic G4[6] strains detected in Korea [15].

The phenomenon that all 20 G4P[6] strains in this study showed the same genotype constellation (G4-P[6]-I1-R1-C1-M1-A1-N1-T1-E1-H1) and high genetic similarities suggested the possibility of persistent infection with the same rotavirus strain over 3 years in one hospital. However, 11 of the 20 G4P[6] rotavirus cases were detected on the first admission day and were transferred from other hospitals or clinics, indicating the occurrence of outside infection because rotavirus infection requires an incubation period for at least 2 days. Additionally, G4P[6] rotavirus infection in Korean neonates has been reported in several studies in other cities in Korea since 1999 [9–13], suggesting that rotavirus G4P[6] infection is not a local phenomenon occurring only at one hospital, but could occur throughout all of South Korea.

The various genotype constellations of the G4P[6] strain are thought to originate from reassortment events of the I5, A8, and T7 genotypes in the original G4-P[6]-I1-R1-C1-M1-A1-N1-T1-E1-H1 genotype constellation (Table 2). The reassortment of rotaviruses is a common phenomenon, and G4P[6] strains are reported to have potentially originated from pigs [8]. Most G4P[6] strains exhibited the Wa-like constellation, whereas G4P[6] strains in Zambia showed the DS-1-like constellation (G4-P6-I2-R2-C2-M2-A2-N2-T2-E2-H2; Table 2; submitted to GenBank: RVA/Human-wt/ZMB/MRC-DPRU1752/XXXX/G4P[6]). The reassortment observed among the 11 rotavirus genes is a relatively common phenomenon in viruses with segmented RNA genes, such as influenza virus [22–24].

Both genotypes of G8P[6] and G4P[6] were frequently detected in Korean neonates, and sequence similarities were observed between P[6]s in G8P[6] strains and P[6]s in G4P[6] strains, whereas differences were found in P[6] sequences from G4P[6] strains detected in other countries. These findings suggested that selective infection by rotaviruses with these unique P[6] sequences occurred in Korean neonates. Moreover, previous reports have demonstrated that the VP8 portion of VP4 attaches to the human blood group antigen (HBGA) in the intestinal epithelium and that there is an association between the antigenicity of VP4 (VP8) and HBGA [25]. Therefore, unique P[6] sequences and the unique antigenicities of G8P[6] and G4P[6] strains may be related to HBGA in the intestinal epithelium in Korean neonates. Further studies are needed to determine the mechanism through which P[6] genotypes easily infect Korean neonates. Current rotavirus vaccination programs (e.g., RotaTeq or Rotarix), which begin after 6 weeks of age, cannot prevent neonatal rotavirus infection [9]. However, a recently developed neonatal rotavirus vaccine (RV3-BB, G3P[6]), which has P[6] antigenicity and is first given 0–5 days after birth, may be effective against Korean neonatal rotavirus G4P[6] infection [26].

## Conclusions

In summary, G4P[6] strains isolated from Korean neonates in 2013–2016 had the same genotype constellation, G4-P[6]-I1-R1-C1-M1-A1-N1-T1-E1-H1 (a Wa-like constellation). Korean G4P[6] and G8P[6] strains have been shown to easily infect Korean neonates, and the common Korean P[6] sequences in G4P[6] and G8P[6] strains have unique nucleotide sequences compared with G4P[6] strains detected in other countries. This may be the cause of the association between P[6] and Korean neonatal rotavirus infection. Further studies are needed to determine the mechanisms through which P[6] genotypes easily infect Korean neonates.

## Methods

### Patient samples

Rotavirus-positive stool samples were collected from neonates younger than 1 month of age in a 650-bed hospital from January 2013 to January 2016. Twenty G4P[6] rotavirus-positive samples were successfully genotyped for whole-gene genotyping using NGS (11 specimens in 2013, six specimens in 2014, two specimens in 2015, one specimen in 2016). During this period, 270 rotavirus antigen-positive samples from neonates with symptomatic diarrhea were collected, and 56 samples were arbitrarily selected for this G4P[6] whole-genome sequencing study. Forty-nine samples from these 56 samples (87.5%) were genotyped as G4P[6] strains using G and P typing (seven samples were non-G4P[6] strains). Of 49 G4P[6] strains, 20 samples were successfully amplified for all 11 rotavirus genes evaluated in whole-genome sequencing. Clinical data, including age and sex, were collected from patient medical records. Eleven (55.0%) samples were collected from males, and the overall median age of the donors was 11 days (range 5–28 days). This study was approved by the Institutional Review Board of Hallym University Dongtan Sacred Heart Hospital (IRB nos. 2013-030, 2017-08-007).

### Whole-genome sequencing of rotaviruses using NGS

Whole-genome sequencing of rotaviruses was carried out using reverse transcription polymerase chain reaction (RT-PCR) and NGS. Viral RNA was extracted from fecal suspensions using a QIAamp Viral RNA Mini kit (Qiagen, Hilden, Germany) and the QIAcube platform (Qiagen). The RNA was denatured and reverse transcribed using the SuperScript III First-Strand Synthesis System (Invitrogen, Carlsbad, CA, USA). Eleven rotavirus genes were amplified from the double-stranded RNA genome using specific primer sets described in Additional file 10: Table S1 [27]. All 20 RT-PCR products for each genome were pooled in equimolar amounts, sheared using an Ion Xpress Plus Fragment Library Kit (Thermo Fisher Scientific, Waltham, MA, USA), and then ligated to barcoded adaptors using Ion Express Barcode Adapter kits (Thermo Fisher Scientific), to create about 300-bp sized fragment libraries. Template preparation, including emulsion PCR, was performed using Ion 510 and Ion 520 and Ion 530 kit-Chef (Thermo Fisher Scientific) and an Ion Chef system (Thermo Fisher Scientific). NGS was performed using the Ion Torrent S5 XL NGS platform (Thermo Fisher Scientific) and Ion S5 Sequencing kit on a 520 chip. Sequenced reads were quality checked and trimmed using Ion Torrent Suite version 5.0.4. Raw sequence data were processed using the CLC genomics workbench (http://www.clcbio.com/). Sequenced reads were trimmed and mapped to the rotavirus reference sequence (ASM265499v1 or ASM268153v1), and consensus sequences of each gene were obtained. Because we could not obtain the sequences of VP7 genes by NGS, VP7 genotyping was carried out using RT-PCR and Sanger sequencing with another specific primer set (46F/911R; Additional file 10: Table S1).

### Rotavirus genotypes and constellation

The genotypes of gene sequences were obtained using the Rota C v2.0 online automated genotyping tool [28], and whole-genome constellations were obtained. The closest nucleotide sequences to each gene were obtained using the Basic Local Alignment Search Tool (BLAST) on the National Center for Biotechnology Information (NCBI) website. Sequence similarities between the genes in this study and other G4P[6] strains with whole-genome sequence data in GenBank were compared using BLAST on the NCBI website.

### Phylogenetic and nucleotide sequence analyses of rotavirus G4P[6] strains

Phylogenetic and nucleotide sequence analyses were performed to investigate genetic distances among rotavirus G4P[6] strains in this study and comparative G4P[6] strains having whole-genome sequences, including RVA/Human tc/GBR/ST3/1975/G4P2A[6], RVA/Human wt/CHN/E931/2008/G4P[6], RVA/Human wt/CHN/GX54/2010/G4P[6], RVA/Human wt/CHN/GX77/2010/G4P[6], RVA/Human wt/CHN/GX82/2010/G4P[6], RVA/Human wt/CHN/R479/2004/G4P[6], RVA/Human wt/CHN/R1954/2013/G4P[6], RVA/Human wt/THA/CMH-N014-11/2011/G4P[6], RVA/Human wt/THA/CMH-N016-10/2010/G4P[6], RVA/Human-wt/LKA/R1207/2009/G4P[6], RVA/Human wt/IND/mani 362/2007/G4P[6], RVA/Human wt/ARG/Arg4605/2006/G4P[6], RVA/Human wt/ARG/Arg4671/2006/G4P[6], RVA/Human wt/HUN/BP271/2000/G4P[6], RVA/Human wt/HUN/BP1125/2004/G4P[6], RVA/Human wt/HUN/BP1227/2002/G4P[6], RVA/Human wt/HUN/BP1231/2002/G4P[6], RVA/Human wt/HUN/BP1490/1994/G4P[6], RVA/Human wt/HUN/BP1547/2005/G4P[6], RVA/Human wt/HUN/BP1792/2004/G4P[6], RVA/Human wt/HUN/BP1901/1991/G4P[6], RVA/Human wt/COD/KisB332/2008/G4P[6], RVA/Human/NCA/OL/2010/G4P[6], RVA/Human wt/PRY/1809SR/2009/G4P[6], RVA/Human-wt/ZMB/MRC-DPRU1752/XXXX/G4P[6], RVA/Human wt/KOR/CAU 195/2006/G12P[6], and RVA/Human wt/KOR/CAU 214/2006/G12P[6]. The previously reported G4 and P[6] sequences of rotavirus G4P[6] strains detected in Korea (KF650074–650095) [10] and the P[6] sequences of rotavirus G8P[6] strains detected in the same Korean hospital in a previous study [15] were also included in the phylogenetic analyses of the VP4 and VP7 genes. The following porcine rotavirus G4 and P[6] sequences of porcine rotavirus G4P[6] strains were included: RVA/Pig-wt/BRA/BRA844/2007/G4P[6], RVA/Pig-tc/Gottfried/1975/G4P[6], RVA/Pig-wt/THA/CULC-1/2013/G4P[6], RVA/Pig-wt/THA/CU-L141/2012/G4P[6], RVA/Pig-wt/THA/CMP-011-09/2009/G4P[6], RVA/Pit-wt/THA/CMP-011-09/2009/G4P[6], RVA/Pig-wt/THA/CULC-1/2013/G4P[6], RVA/Pig-wt/WNM/14249 23/G4P[6], and RVA/Pig-wt/VNM/12129 49/G4P[6]. Reference sequences of rotaviruses were obtained from the NCBI virus genome resource (https://www.ncbi.nlm.nih.gov/genome/viruses/variation/). MEGA software version 7 was used for phylogenetic analysis [29]. Phylogenetic trees were constructed using the maximum likelihood method and Tamura–Nei substitution models with 1000 bootstrap replicates.

## Nucleotide sequence accession numbers

We submitted the rotavirus sequences in our study to the GenBank and obtained the GenBank accession numbers for the nucleotide sequences of the 11 genes of strains RN-001, RN-010, RN-014, RN-017, and RN-019, respectively: MK953602 (RN-001_VP1), MK953603 (RN-001_VP2), MK953604 (RN-001_VP3), MK953605 (RN-001_VP4), MK953606 (RN-001_VP6), MK953607 (RN-001_VP7), MK953597 (RN-001_NSP1), MK953598 (RN-001_NSP2), MK953599 (RN-001_NSP3), MK953600 (RN-001_NSP4), MK953601 (RN-001_NSP5/6), MK953584 (RN-010_VP1), MK953589 (RN-010_VP2), MK953583 (RN-010_VP3), MK953582 (RN-010_VP4), MK953581 (RN-010_VP6), MK953580 (RN-010_VP7), MK953590 (RN-010_NSP1), MK953588 (RN-010_NSP2), MK953587 (RN-010_NSP3), MK953586 (RN-010_NSP4), MK953585 (RN-010_NSP5/6), MK953575 (RN-014_VP1), MK953591 (RN-014_VP2), MK953574 (RN-014_VP3), MK953573 (RN-014_VP4), MK953572 (RN-014_VP6), MK953571 (RN-014_VP7), MK953592 (RN-014_NSP1), MK953579 (RN-014_NSP2), MK953578 (RN-014_NSP3), MK953577 (RN-014_NSP4), MK953576 (RN-014_NSP5/6), MK953566 (RN-017_VP1), MK953593 (RN-017_VP2), MK953565 (RN-017_VP3), MK953564 (RN-017_VP4), MK953563 (RN-017_VP6), MK953562 (RN-017_VP7), MK953594 (RN-017_NSP1), MK953570 (RN-017_NSP2), MK953569 (RN-017_NSP3), MK953568 (RN-017_NSP4), MK953567 (RN-017_NSP5/6), MK953557 (RN-019_VP1), MK953595 (RN-019_VP2), MK953556 (RN-019_VP3), MK953555 (RN-019_VP4), MK953554 (RN-019_VP6), MK953553 (RN-019_VP7), MK953596, (RN-019_NSP1), MK953561 (RN-019_NSP2), MK953560 (RN-019_NSP3), MK953559 (RN-019_NSP4), MK953558 (RN-019_NSP5/6).

## Additional files

**Additional file 1: Fig. S1.** Phylogenetic tree of VP6 (I1) sequences of rotavirus G4P[6] strains in this study and other G4P[6] rotavirus strains with full genome sequences. Black circles indicate the G4P[6] strains isolated from neonates in this study, and black diamonds indicate the Korean G4P[6] strains from GenBank.

**Additional file 2: Fig. S2.** Phylogenetic tree of VP1 (R1) sequences of rotavirus G4P[6] strains in this study and other G4P[6] rotavirus strains with full genome sequences. Black circles indicate the G4P[6] strains isolated from neonates in this study, and black diamonds indicate the Korean G4P[6] strains from GenBank.

**Additional file 3: Fig. S3.** Phylogenetic tree of VP2 (C1) sequences of rotavirus G4P[6] strains in this study and other G4P[6] rotavirus strains with full genome sequences. Black circles indicate the G4P[6] strains isolated from neonates in this study, and black diamonds indicate the Korean G4P[6] strains from GenBank.

**Additional file 4: Fig. S4.** Phylogenetic tree of VP3 (M1) sequences of rotavirus G4P[6] strains in this study and other G4P[6] rotavirus strains with full genome sequences. Black circles indicate the G4P[6] strains isolated from neonates in this study, and black diamonds indicate the Korean G4P[6] strains from GenBank.

**Additional file 5: Fig. S5.** Phylogenetic tree of NSP1 (A1) sequences of rotavirus G4P[6] strains in this study and other G4P[6] rotavirus strains with full genome sequences. Black circles indicate the G4P[6] strains isolated from neonates in this study, and black diamonds indicate the Korean G4P[6] strains from GenBank.

**Additional file 6: Fig. S6.** Phylogenetic tree of NSP2 (N1) sequences of rotavirus G4P[6] strains in this study and other G4P[6] rotavirus strains with full genome sequences. Black circles indicate the G4P[6] strains isolated from neonates in this study, and black diamonds indicate the Korean G4P[6] strains from GenBank.

**Additional file 7: Fig. S7.** Phylogenetic tree of NSP3 (T1) sequences of rotavirus G4P[6] strains in this study and other G4P[6] rotavirus strains with full genome sequences. Black circles indicate the G4P[6] strains isolated from neonates in this study, and black diamonds indicate the Korean G4P[6] strains from GenBank.

**Additional file 8: Fig. S8.** Phylogenetic tree of NSP4 (E1) sequences of rotavirus G4P[6] strains in this study and other G4P[6] rotavirus strains with full genome sequences. Black circles indicate the G4P[6] strains isolated from neonates in this study, and black diamonds indicate the Korean G4P[6] strains from GenBank.

**Additional file 9: Fig. S9.** Phylogenetic tree of NSP5/6 (H1) sequences of rotavirus G4P[6] strains in this study and other G4P[6] rotavirus strains with full genome sequences. Black circles indicate the G4P[6] strains isolated from neonates in this study, and black diamonds indicate the Korean G4P[6] strains from GenBank.

**Additional file 10: Table S1.** Primers used in this study [27].

## Data Availability

The datasets used and/or analyzed during the current study are available from the corresponding author on reasonable request.
