# Supplementary figures and images for: Whole-genome analysis of rotavirus G4P[6] strains isolated from Korean neonates: association of Korean neonates and rotavirus P[6] genotypes
Source: Gut Pathog. 2019 Jul 10;11:37. doi: 10.1186/s13099-019-0318-5 (PMC6621965; doi:10.1186/s13099-019-0318-5)

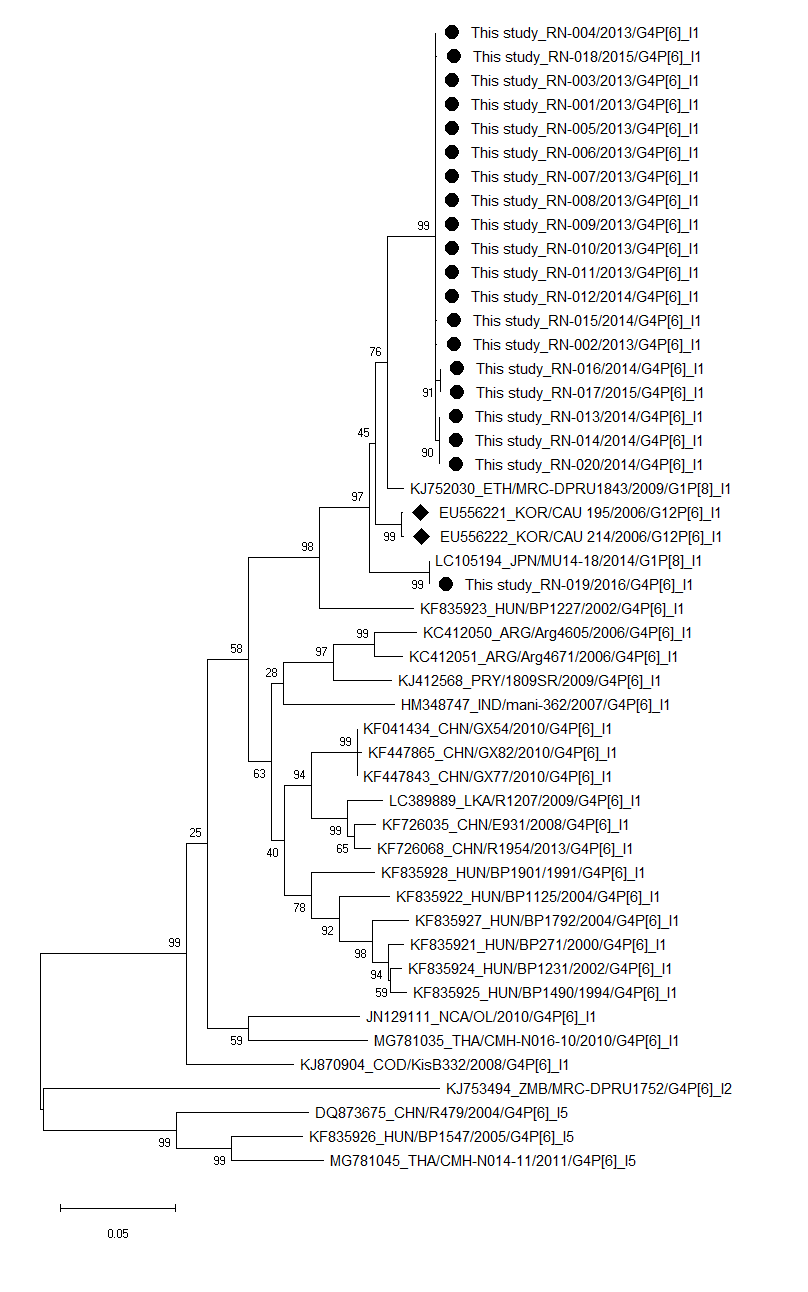

Supplement: Supplementary file 1 — Additional file 1: Fig. S1. Phylogenetic tree of VP6 (I1) sequences of rotavirus G4P[6] strains in this study and other G4P[6] rotavirus strains with full genome sequences. Black circles indicate the G4P[6] strains isolated from neonates in this study, and black diamonds indicate the Korean G4P[6] strains from GenBank. [file 13099_2019_318_MOESM1_ESM.tif]

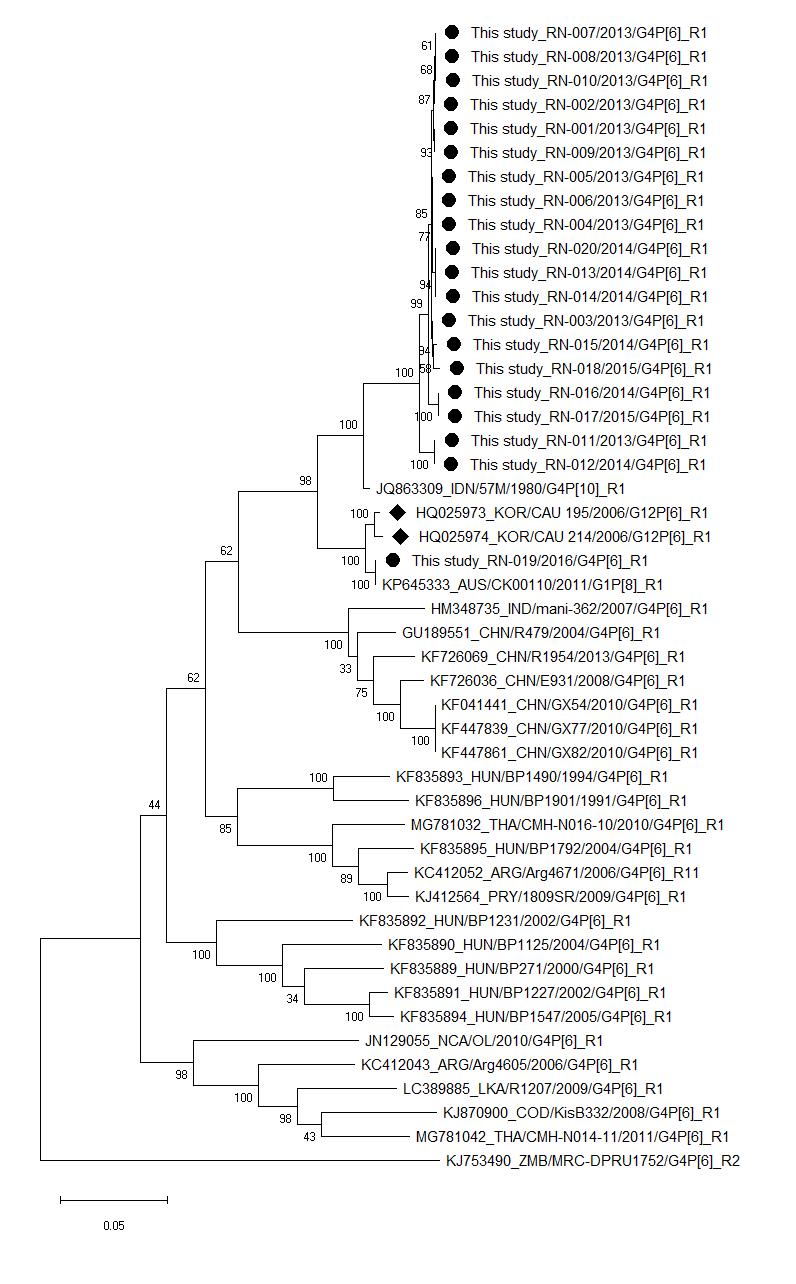

Supplement: Supplementary file 2 — Additional file 2: Fig. S2. Phylogenetic tree of VP1 (R1) sequences of rotavirus G4P[6] strains in this study and other G4P[6] rotavirus strains with full genome sequences. Black circles indicate the G4P[6] strains isolated from neonates in this study, and black diamonds indicate the Korean G4P[6] strains from GenBank. [file 13099_2019_318_MOESM2_ESM.tif]

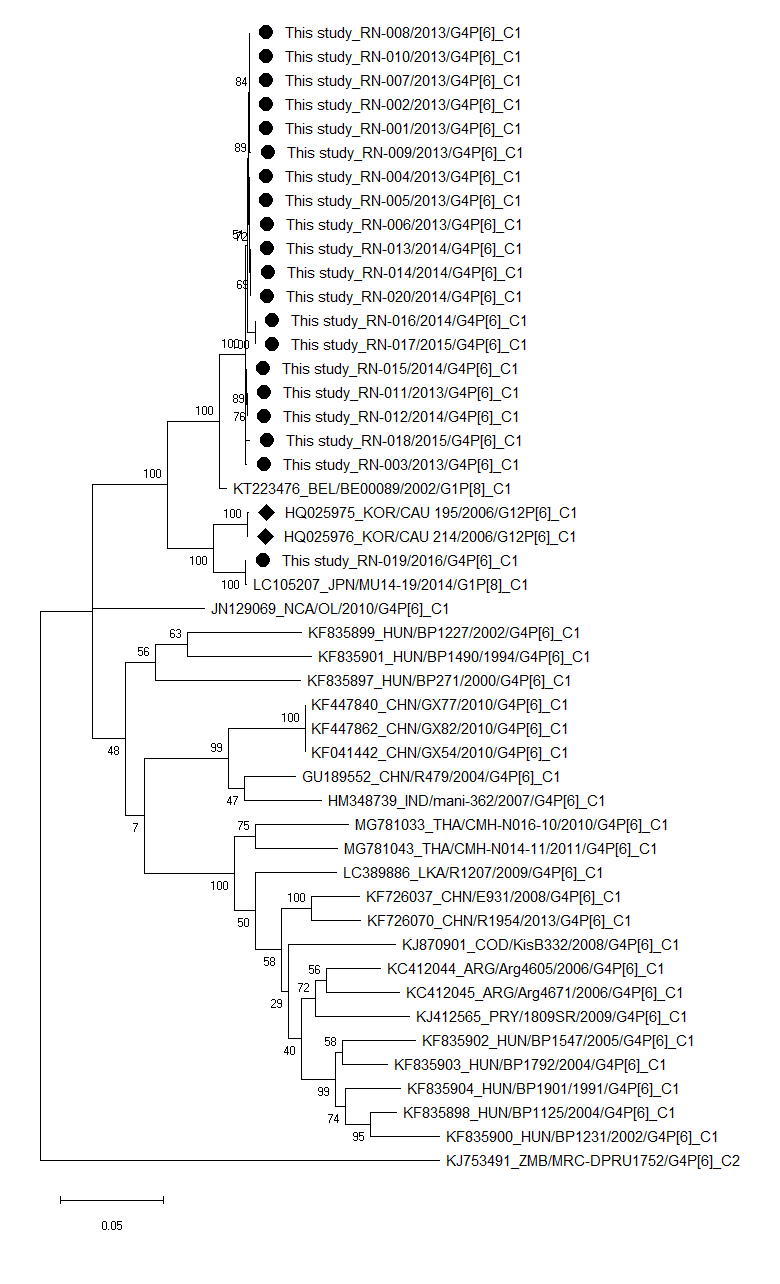

Supplement: Supplementary file 3 — Additional file 3: Fig. S3. Phylogenetic tree of VP2 (C1) sequences of rotavirus G4P[6] strains in this study and other G4P[6] rotavirus strains with full genome sequences. Black circles indicate the G4P[6] strains isolated from neonates in this study, and black diamonds indicate the Korean G4P[6] strains from GenBank. [file 13099_2019_318_MOESM3_ESM.tif]

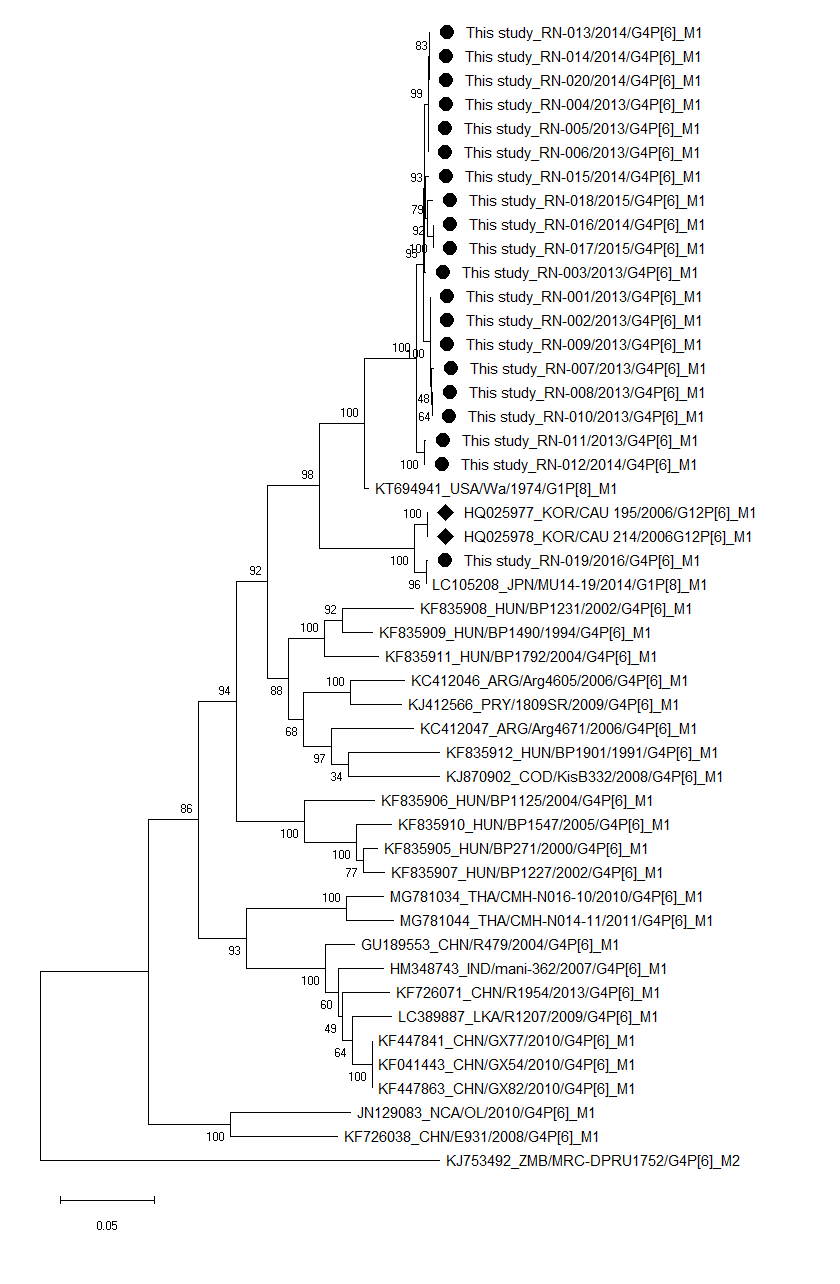

Supplement: Supplementary file 4 — Additional file 4: Fig. S4. Phylogenetic tree of VP3 (M1) sequences of rotavirus G4P[6] strains in this study and other G4P[6] rotavirus strains with full genome sequences. Black circles indicate the G4P[6] strains isolated from neonates in this study, and black diamonds indicate the Korean G4P[6] strains from GenBank. [file 13099_2019_318_MOESM4_ESM.tif]

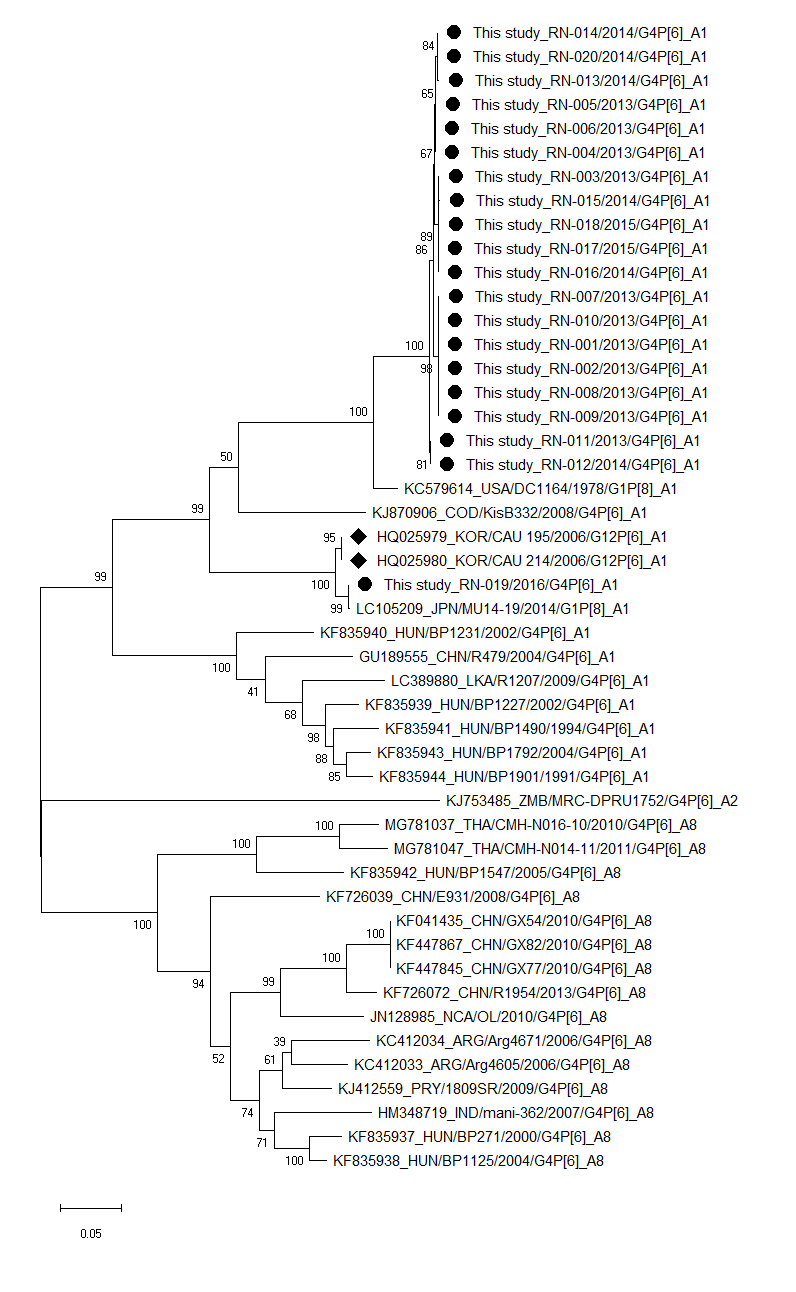

Supplement: Supplementary file 5 — Additional file 5: Fig. S5. Phylogenetic tree of NSP1 (A1) sequences of rotavirus G4P[6] strains in this study and other G4P[6] rotavirus strains with full genome sequences. Black circles indicate the G4P[6] strains isolated from neonates in this study, and black diamonds indicate the Korean G4P[6] strains from GenBank. [file 13099_2019_318_MOESM5_ESM.tif]

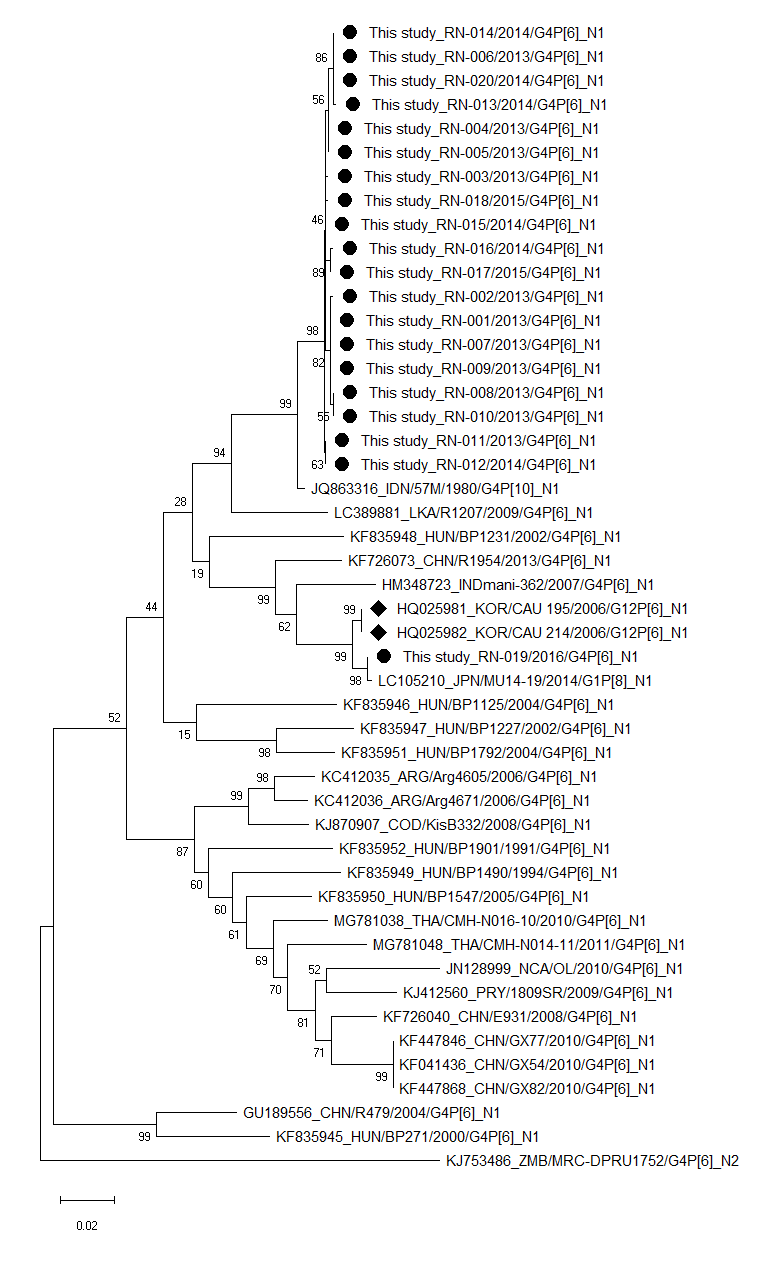

Supplement: Supplementary file 6 — Additional file 6: Fig. S6. Phylogenetic tree of NSP2 (N1) sequences of rotavirus G4P[6] strains in this study and other G4P[6] rotavirus strains with full genome sequences. Black circles indicate the G4P[6] strains isolated from neonates in this study, and black diamonds indicate the Korean G4P[6] strains from GenBank. [file 13099_2019_318_MOESM6_ESM.tif]

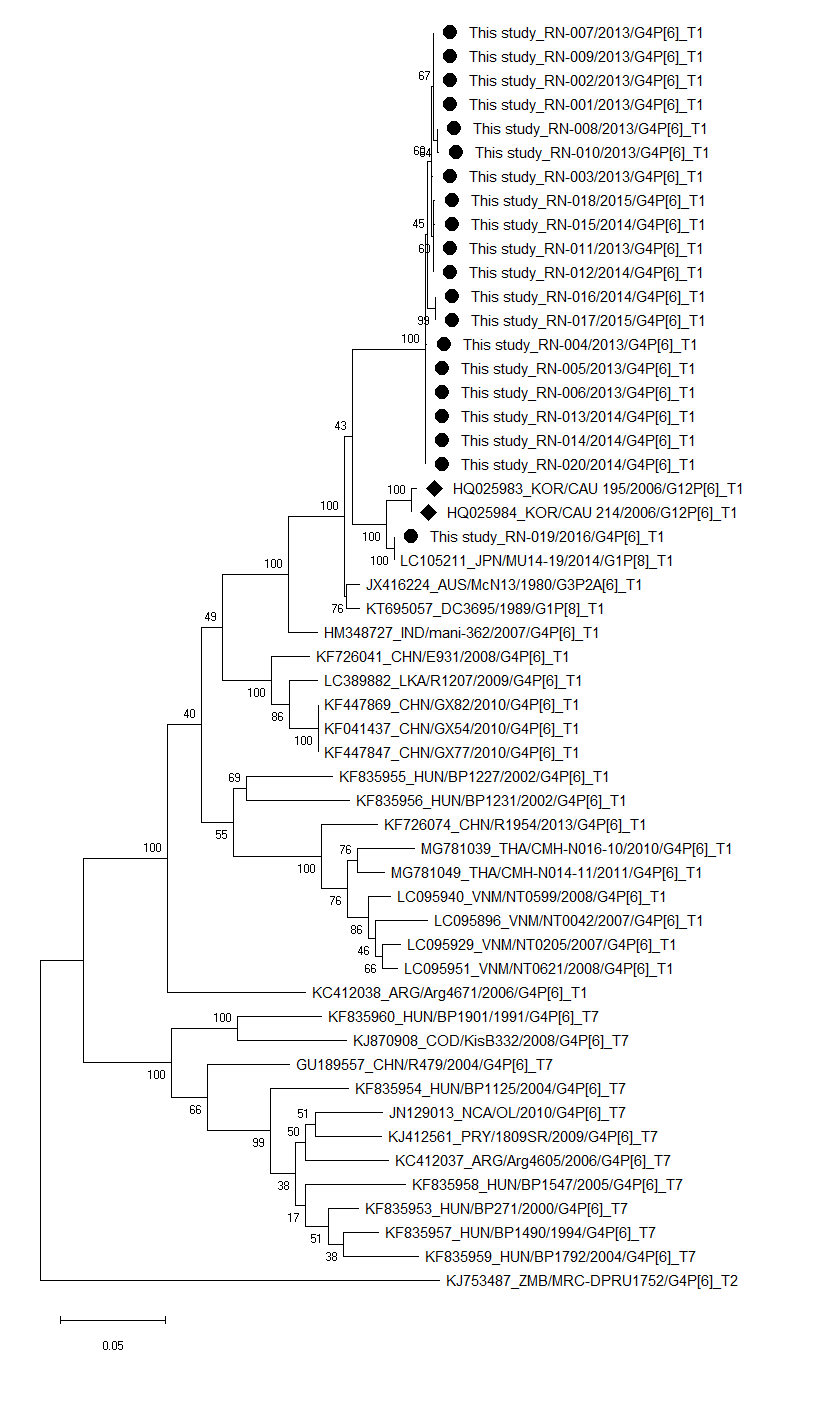

Supplement: Supplementary file 7 — Additional file 7: Fig. S7. Phylogenetic tree of NSP3 (T1) sequences of rotavirus G4P[6] strains in this study and other G4P[6] rotavirus strains with full genome sequences. Black circles indicate the G4P[6] strains isolated from neonates in this study, and black diamonds indicate the Korean G4P[6] strains from GenBank. [file 13099_2019_318_MOESM7_ESM.tif]

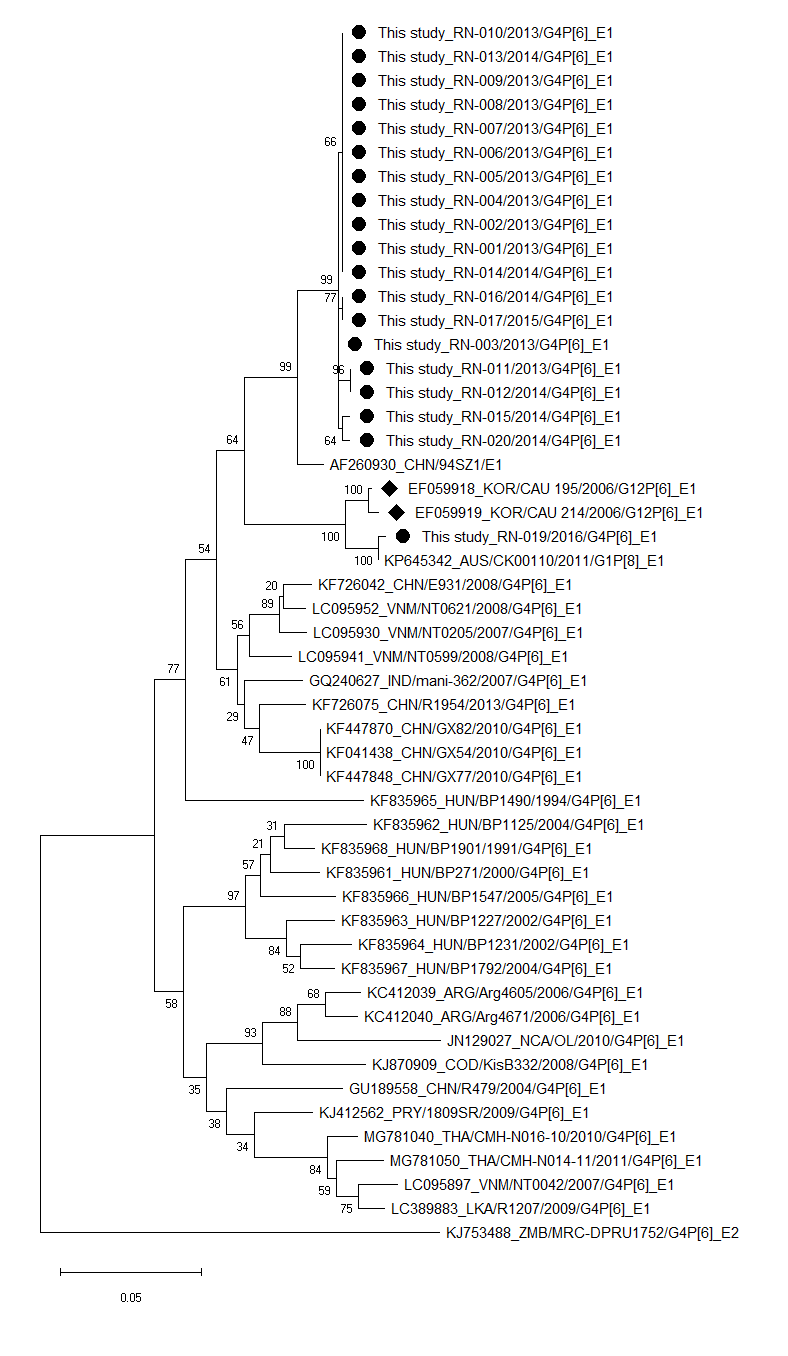

Supplement: Supplementary file 8 — Additional file 8: Fig. S8. Phylogenetic tree of NSP4 (E1) sequences of rotavirus G4P[6] strains in this study and other G4P[6] rotavirus strains with full genome sequences. Black circles indicate the G4P[6] strains isolated from neonates in this study, and black diamonds indicate the Korean G4P[6] strains from GenBank. [file 13099_2019_318_MOESM8_ESM.tif]

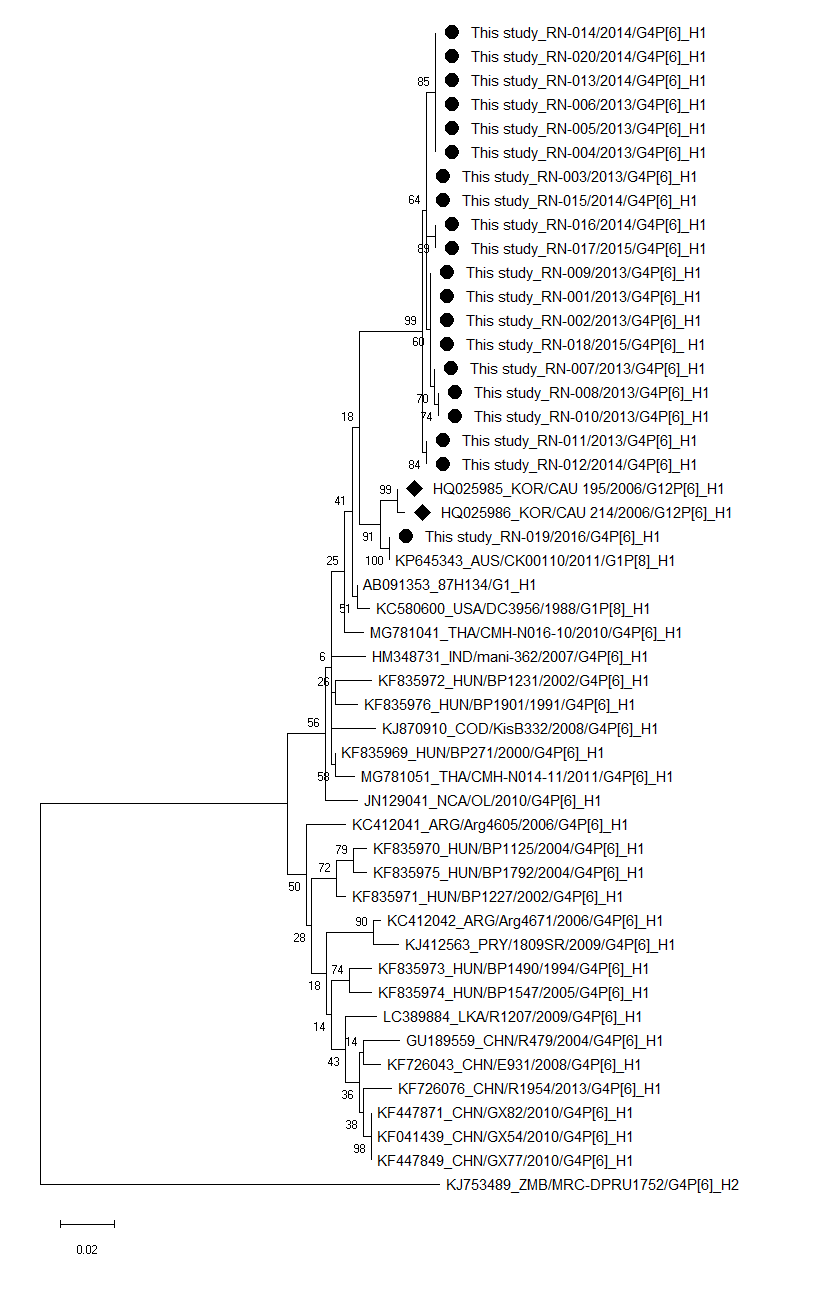

Supplement: Supplementary file 9 — Additional file 9: Fig. S9. Phylogenetic tree of NSP5/6 (H1) sequences of rotavirus G4P[6] strains in this study and other G4P[6] rotavirus strains with full genome sequences. Black circles indicate the G4P[6] strains isolated from neonates in this study, and black diamonds indicate the Korean G4P[6] strains from GenBank. [file 13099_2019_318_MOESM9_ESM.tif]
