# Supplementary material for: Whole-genome analysis of rotavirus G4P[6] strains isolated from Korean neonates: association of Korean neonates and rotavirus P[6] genotypes
Source: Gut Pathog. 2019 Jul 10;11:37. doi: 10.1186/s13099-019-0318-5 (PMC6621965; doi:10.1186/s13099-019-0318-5)
Supplement: Supplementary file 10 — Additional file 10: Table S1. Primers used in this study [27]. [file 13099_2019_318_MOESM10_ESM.docx]

**Additional file 10.** Table S1. Primers used in this study [27].

| Gene | Primers | Sequence (5’-3’) | Amplicon size (bp) |
| --- | --- | --- | --- |
| VP4 | 1F  2359R | TGTAAAACGACGGCCAGTGGCTATAAAATGGCTTC-  CAGGAAACAGCTATGACCGGTCACATCCTCAATAG | 2359 |
| VP7 | 1F  1063R | TGTAAAACGACGGCCAGTGGCTTTAAAAGAGAGAATTTC  CAGGAAACAGCTATGACCGGTCACATCRWACAATTC | 1063 |
| VP7 | 46F  911R | TTAATGTATGGTATTGAATA  TCACTCGCATCATTCGTTCC | 866 |
| VP6 | 1F  1364R | TGTAAAACGACGGCCAGTGGCTTTWAAACGAAGTCTTC  CAGGAAACAGCTATGACCGGTCACATCCTCTCAC | 1364 |
| VP1 | 1F  3302R | TGTAAAACGACGGCCAGTGGCTATTAAAGCTGTAC  CAGGAAACAGCTATGACCGGTCACATCTAAGCAC | 3302 |
| VP2 | 1F  2723R | TGTAAAACGACGGCCAGTGGCTATTAAAGGCTCAATG  CAGGAAACAGCTATGACCGGTCATATCTCCACAGTG | 2723 |
| VP3 | 24F  2591R | TGTAAAACGACGGCCAGTGTTTTACCTCTGATGGTG  CAGGAAACAGCTATGACCGGTCACATCATGACTAG | 2568 |
| NSP1 | 9F  1565R | TGTAAAACGACGGCCAGTTTTGAAAAGTCTTGTGGAAG  CAGGAAACAGCTATGACCGGTCACATTTTATGCTGC | 1557 |
| NSP2 | 1F  1050R | TGTAAAACGACGGCCAGTCGTCTCAGTCGCCGTTTG  CAGGAAACAGCTATGACCAGCGCTTTCTATTCTTRC | 1050 |
| NSP3 | 1F  1055R | TGTAAAACGACGGCCAGTGGCWTTTAATGCTTTTCAG  CAGGAAACAGCTATGACCCCATTTAGGTTTTTGACAG | 1055 |
| NSP4 | 1F  748R | TGTAAAACGACGGCCAGTGTTCTGTTCCGAGAGAGC  CAGGAAACAGCTATGACCTCACRYTAAGACCRTTCC | 748 |
| NSP5/6 | 1F  673R | TGTAAAACGACGGCCAGTGGCTTTWAAAGCGCTAC  CAGGAAACAGCTATGACCGGTCACAAAACGGGAG | 673 |
